# Supplementary material for: Marine catfishes (Ariidae—Siluriformes) from the Coastal Amazon: mitochondrial DNA barcode for a recent diversification group?
Source: PeerJ. 2024 Aug 28;12:e17581. doi: 10.7717/peerj.17581 (PMC11365480; doi:10.7717/peerj.17581)
Supplement: Supplemental Information 1 [file peerj-12-17581-s001.docx]

**Supplementary Material 1.** Haplotype generation of Ariidae diversity in the Coastal Amazon.

**COI**

Number of sequences used: 57

Number of haplotypes, h: 25

Haplotype diversity, Hd: 0,9586

Hap_1: 5 [*Notarius phrygiatus* 4; *Notarius phrygiatus* 6; *Notarius phrygiatus* 7; *Notarius quadriscutis* 4; *Notarius quadriscutis* 5]

Hap_2: 5 [*Notarius rugispinis* 1; *Notarius rugispinis* 2; *Notarius rugispinis* 3; *Notarius rugispinis* 4; *Notarius rugispinis* 5]

Hap_3: 2 [*Notarius quadriscutis* 1; *Notarius quadriscutis* 3]

Hap_4: 1 [*Notarius quadriscutis* 2]

Hap_5: 2 [*Bagre bagre* 2; *Bagre bagre* 4]

Hap_6: 2 [*Bagre bagre* 3; *Bagre bagre* 5]

Hap_7: 1 [*Bagre bagre* 6]

Hap_8: 1 [*Cathorops agassizii* 4]

Hap_9: 3 [*Cathorops agassizii* 7; *Cathorops agassizii* 8; *Cathorops agassizii* 10]

Hap_10: 1 [*Cathorops spixii* 1]

Hap_11: 1 [*Cathorops spixii* 2]

Hap_12: 2 [*Cathorops spixii* 3; *Cathorops spixii* 5]

Hap_13: 1 [*Cathorops spixii* 4]

Hap_14: 2 [*Notarius grandicassis* 1; *Notarius grandicassis* 2]

Hap_15: 2 [*Notarius grandicassis* 3; *Notarius grandicassis* 4]

Hap_16: 1 [*Notarius grandicassis* 5]

Hap_17: 1 [*Sciades couma* 1]

Hap_18: 3 [*Sciades couma* 2; *Sciades couma* 4; *Sciades couma* 6]

Hap_19: 1 [*Sciades couma* 3]

Hap_20: 1 [*Sciades couma* 5]

Hap_21: 5 [*Sciades herzbergii* 1; *Sciades herzbergii* 2; *Sciades herzbergii* 3; *Sciades herzbergii* 4; *Sciades herzbergii* 5]

Hap_22: 6 [*Sciades parkeri* 3; *Sciades parkeri* 4; *Sciades parkeri* 5; *Sciades parkeri* 6; *Sciades parkeri* 7; *Sciades parkeri* 8]

Hap_23: 4 [*Sciades passany* 1; *Sciades passany* 2; *Sciades passany* 3; *Sciades passany* 5]

Hap_24: 3 [*Sciades proops* 1; *Sciades proops* 4; *Sciades proops* 5]

Hap_25: 1 [*Sciades proops* 3]

**Cytb**

Number of sequences used: 57

Number of haplotypes, h: 31

Haplotype diversity, Hd: 0,9668

Hap_1: 2 [*Notarius phrygiatus* 4; *Notarius quadriscutis* 4]

Hap_2: 1 [*Notarius phrygiatus* 6]

Hap_3: 2 [*Notarius phrygiatus* 7; *Notarius quadriscutis* 2]

Hap_4: 5 [*Notarius rugispinis* 1; *Notarius rugispinis* 2; *Notarius rugispinis* 3; *Notarius rugispinis* 4; *Notarius rugispinis* 5]

Hap_5: 1 [*Notarius quadriscutis* 1]

Hap_6: 1 [*Notarius quadriscutis* 3]

Hap_7: 1 [*Notarius quadriscutis* 5]

Hap_8: 1 [*Bagre bagre* 2]

Hap_9: 1 [*Bagre bagre* 3]

Hap_10: 1 [*Bagre bagre* 4]

Hap_11: 1 [*Bagre bagre* 5]

Hap_12: 1 [*Bagre bagre* 6]

Hap_13: 4 [*Cathorops agassizii* 4; *Cathorops agassizii* 7; *Cathorops agassizii* 8; *Cathorops agassizii* 10]

Hap_14: 1 [*Cathorops spixii* 1]

Hap_15: 1 [*Cathorops spixii* 2]

Hap_16: 1 [*Cathorops spixii* 3]

Hap_17: 2 [*Cathorops spixii* 4; *Cathorops spixii* 5]

Hap_18: 3 [*Notarius grandicassis* 1; *Notarius grandicassis* 3; *Notarius grandicassis* 4]

Hap_19: 1 [*Notarius grandicassis* 2]

Hap_20: 1 [*Notarius grandicassis* 5]

Hap_21: 1 [*Sciades couma* 1]

Hap_22: 1 [*Sciades couma* 2]

Hap_23: 3 [*Sciades couma* 3; *Sciades couma* 5; *Sciades couma* 6]

Hap_24: 1 [*Sciades couma* 4]

Hap_25: 2 [*Sciades herzbergii* 1; *Sciades herzbergii* 5]

Hap_26: 1 [*Sciades herzbergii* 2]

Hap_27: 1 [*Sciades herzbergii* 3]

Hap_28: 1 [*Sciades herzbergii* 4]

Hap_29: 6 [*Sciades parkeri* 3; *Sciades parkeri* 4; *Sciades parkeri* 5; *Sciades parkeri* 6; *Sciades parkeri* 7; *Sciades*_*parkeri* 8]

Hap_30: 4 [*Sciades passany* 1; *Sciades passany* 2; *Sciades passany* 3; *Sciades passany* 5]

Hap_31: 4 [*Sciades proops* 1; *Sciades proops* 3; *Sciades proops* 4; *Sciades proops* 5]
