# Supplementary material for: Marine catfishes (Ariidae—Siluriformes) from the Coastal Amazon: mitochondrial DNA barcode for a recent diversification group?
Source: PeerJ. 2024 Aug 28;12:e17581. doi: 10.7717/peerj.17581 (PMC11365480; doi:10.7717/peerj.17581)
Supplement: Supplemental Information 2 — The numbering after each scientific name represents species individuals. GB = GenBank–National Center for Biotechnology Information; BOLD = Barcode of Life Data System. [file peerj-12-17581-s002.docx]

**Supplementary Material 2.** Comparative list of Ariidae haplotypes from the coastal Amazon region from the COI mitochondrial gene and public databases. The numbering after each scientific name represents species individuals. GB = GenBank – National Center for Biotechnology Information; BOLD = Barcode of Life Data System.

| **Haplotype code and frequency of occurrence** | **Haplotype**  **(based on morphology)** | **Species identified and GB deposit number** | **Similarity GB** | **Identified species and BOLD filing number** | **Similarity BOLD** |
| --- | --- | --- | --- | --- | --- |
| H1 (5) | *Notarius phrygiatus* 4 | *Notarius luniscutis*  (KY402450.1) | 100% | *Notarius grandicassis*  (MFSP682-10) | 100% |
| H2 (2) | *Notarius quadriscutis* 1 | *Notarius luniscutis*  (KY402450.1) | 99.8% | *Notarius grandicassis*  (MFSP685-10) | 99.79% |
| H3 (1) | *Notarius quadriscutis* 2 | *Notarius luniscutis*  (KY402450.1) | 99.8% | *Notarius grandicassis*  (MFSP685-10) | 99.79% |
| H4 (2) | *Notarius grandicassis* 1 | No match | - | *Notarius grandicassis*  (Private) | 100% |
| H5 (2) | *Notarius grandicassis* 3 | No match | - | *Notarius grandicassis*  (Private) | 99.8% |
| H6 (1) | *Notarius grandicassis* 5 | No match | - | *Notarius grandicassis*  (Private) | 100% |
| H7 (5) | *Notarius rugispinis* 1 | No match | - | *Notarius rugispinis*  (ITAPE389-15) | 100% |
| H8 (1) | *Cathorops agassizii* 4 | *Cathorops spixii*  (OQ075616.1) | 99.37% | *Cathorops spixii*  (GBMNB4209-20) | 99% |
| H9 (3) | *Cathorops agassizii* 7 | *Cathorops spixii*  (OQ075616.1) | 100% | *Cathorops spixii*  (GBMNB4209-20) | 98.8% |
| H10 (1) | *Cathorops spixii* 1 | *Cathorops spixii*  (MF595235.1) | 99.6% | *Cathorops spixii*  (GBMNB4209-20) | 99.6% |
| H11 (1) | *Cathorops spixii* 2 | *Cathorops spixii*  (MF595235.1) | 99.8% | *Cathorops spixii*  (GBMNB4209-20) | 99.79% |
| H12 (2) | *Cathorops spixii* 3 | *Cathorops spixii*  (MF595235.1) | 100% | *Cathorops spixii*  (GBMNB4209-20) | 100% |
| H13 (1) | *Cathorops spixii* 4 | *Cathorops spixii*  (MF595235.1) | 100% | *Cathorops spixii*  (GBMNB4209-20) | 99.79% |
| H14 (3) | *Sciades proops* 1 | *Sciades proops*  (OR195675.1) | 98.99% | *Sciades proops*  (Private) | 99.59% |
| H15 (1) | *Sciades proops* 3 | *Sciades proops*  (OR195675.1) | 99.5% | *Sciades proops*  (Private) | 99.79% |
| H16 (2) | *Bagre bagre* 2 | *Bagre bagre*  (KC618665.1) | 99.8% | *Bagre bagre*  (ANGBF24329-19) | 99.79% |
| H17 (2) | *Bagre bagre* 3 | *Bagre bagre*  (KC618665.1) | 100% | *Bagre bagre*  (ANGBF24329-19) | 100% |
| H18 (1) | *Bagre bagre* 6 | *Bagre bagre*  (KC618665.1) | 99.8% | *Bagre bagre*  (ANGBF24329-19) | 99.79% |
| H19 (1) | *Sciades couma* 1 | No match | - | *Sciades couma*  (ITAPE022-15) | 99.79% |
| H20 (3) | *Sciades couma* 2 | No match | - | *Sciades couma*  (ITAPE022-15) | 100% |
| H21 (1) | *Sciades couma* 3 | No match | - | *Sciades couma*  (ITAPE022-15) | 99.59% |
| H22 (1) | *Sciades couma* 5 | No match | - | *Sciades couma*  (ITAPE022-16) | 99.79% |
| H23 (5) | *Sciades herzbergii* 1 | *Sciades herzbergii*  (OQ075619.1) | 100% | *Sciades herzbergii*  (Private) | 100% |
| H24 (6) | *Sciades parkeri* 3 | *Netuma* sp.  (HQ689375.1) | 100% | *Sciades parkeri*  (Private) | 100% |
| H25 (4) | *Sciades passany* 1 | No match | - | *Sciades passany*  (Private) | 100% |
