# Supplementary material for: Marine catfishes (Ariidae—Siluriformes) from the Coastal Amazon: mitochondrial DNA barcode for a recent diversification group?
Source: PeerJ. 2024 Aug 28;12:e17581. doi: 10.7717/peerj.17581 (PMC11365480; doi:10.7717/peerj.17581)
Supplement: Supplemental Information 3 — The numbering after each scientific name represents species individuals. GB = GenBank-National Center for Biotechnology Information. [file peerj-12-17581-s003.docx]

**Supplementary Material 3.** Comparative list of Ariidae haplotypes from the coastal Amazon region from the Cytb mitochondrial gene and public database. The numbering after each scientific name represents species individuals. GB = GenBank - National Center for Biotechnology Information.

| **Haplotype code and frequency of occurrence** | **Haplotype**  **(based on morphology)** | **Species identified and GB deposit number** | **Similarity GB** |
| --- | --- | --- | --- |
| H1 (2) | *Notarius phrygiatus* 4 | *Notarius quadriscutis* (AY688670.1) */ Notarius luniscutis* (MH324935.1) | 99.72% / 99.51% |
| H2 (1) | *Notarius phrygiatus* 6 | *Notarius luniscutis* (FJ626172.1) */ Notarius quadriscutis* (AY688670.1) | 99.71% / 99.43% |
| H3 (2) | *Notarius phrygiatus* 7 | *Notarius luniscutis* (FJ626172.1) */ Notarius quadriscutis* (AY688670.1) | 99.57% / 99.29% |
| H4 (5) | *Notarius rugispinis* 1 | *Notarius rugispinis*  (AY688668.1) | 100% |
| H5 (1) | *Notarius quadriscutis* 1 | *Notarius luniscutis* (MH324935.1) */ Notarius quadriscutis* (MH340487.1) | 99.67% / 99.67% |
| H6 (1) | *Notarius quadriscutis* 3 | *Notarius luniscutis* (MH324935.1) */ Notarius quadriscutis* (MH340487.1) | 99.34% / 99.34% |
| H7 (1) | *Notarius quadriscutis* 5 | *Notarius luniscutis* (MH324935.1) */ Notarius quadriscutis* (MH340487.1) | 99.51% / 99.51% |
| H8 (1) | *Bagre bagre* 2 | *Bagre bagre*  (KU668613.1) | 100% |
| H9 (1) | *Bagre bagre* 3 | *Bagre bagre*  (KU668614.1) | 100% |
| H10 (1) | *Bagre bagre* 4 | *Bagre bagre*  (KU668614.1) | 99.84% |
| H11 (1) | *Bagre bagre* 5 | *Bagre bagre*  (AY688673.1) | 100% |
| H12 (1) | *Bagre bagre* 6 | *Bagre bagre* (KU668613.1) | 99.34% |
| H13 (4) | *Cathorops agassizii* 4 | *Cathorops spixii* (DQ990474.1) */ Cathorops arenatus* (DQ990475.1) | 99.72% / 98.44% |
| H14 (1) | *Cathorops spixii* 1 | *Cathorops arenatus* (DQ990475.1) */ Cathorops spixii* (DQ990474.1) | 99.72% / 98.44% |
| H15 (1) | *Cathorops spixii* 2 | *Cathorops arenatus* (DQ990475.1) */ Cathorops spixii* (DQ990474.1) | 99.86% / 98.3% |
| H16 (1) | *Cathorops spixii* 3 | *Cathorops arenatus* (DQ990475.1) */ Cathorops spixii* (DQ990474.1) | 100% / 98.44% |
| H17 (2) | *Cathorops spixii* 4 | *Cathorops arenatus* (DQ990475.1) */ Cathorops spixii* (DQ990474.1) | 99.57% / 98.3% |
| H18 (3) | *Notarius grandicassis* 1 | *Notarius grandicassis*  (KX099399.1) | 100% |
| H19 (1) | *Notarius grandicassis* 2 | *Notarius grandicassis*  (KX099399.1) | 99.83% |
| H20 (1) | *Notarius grandicassis* 5 | *Notarius grandicassis*  (KX099395.1) | 100% |
| H21 (1) | *Sciades couma* 1 | *Sciades couma*  (MH733618.1) | 99.72% |
| H22 (1) | *Sciades couma* 2 | *Sciades couma*  (MH733618.1) | 99.29% |
| H23 (3) | *Sciades couma* 3 | *Sciades couma*  (MH733618.1) | 99.43% |
| H24 (1) | *Sciades couma* 4 | *Sciades couma*  (MH733618.1) | 99.15% |
| H25 (2) | *Sciades herzbergii* 1 | *Sciades herzbergii*  (DQ990496.1) | 99.29% |
| H26 (1) | *Sciades herzbergii* 2 | *Sciades herzbergii*  (DQ990496.1) | 99.14% |
| H27 (1) | *Sciades herzbergii* 3 | *Sciades herzbergii*  (DQ990496.1) | 99.15% |
| H28 (1) | *Sciades herzbergii* 4 | *Sciades herzbergii*  (DQ990496.1) | 99.14% |
| H29 (6) | *Sciades parkeri* 3 | *Sciades parkeri*  (DQ990492.1) | 100% |
| H30 (4) | *Sciades passany* 1 | *Sciades passany*  (DQ990493.1) | 100% |
| H31 (4) | *Sciades proops* 1 | *Sciades proops*  (DQ990490.1) | 100% |
