# Supplementary material for: Marine catfishes (Ariidae—Siluriformes) from the Coastal Amazon: mitochondrial DNA barcode for a recent diversification group?
Source: PeerJ. 2024 Aug 28;12:e17581. doi: 10.7717/peerj.17581 (PMC11365480; doi:10.7717/peerj.17581)
Supplement: Supplemental Information 4 [file peerj-12-17581-s004.docx]

**Supplementary Material 4.** Interspecific and intraspecific genetic divergences, based on fragments of the COI and Cytb mitochondrial genes for Ariidae species from the coastal region of the Amazon, using corrected distance (K2P).

| **Species** |  | **COI** |  |  | **Cytb** |  |
| --- | --- | --- | --- | --- | --- | --- |
|  | **Interspecific** | **Intraspecific** | ***Barcoding gap*** | **Interspecific** | **Intraspecific** | ***Barcoding gap*** |
| *Notarius phrygiatus* | 0.000-0.158 | 0.000 | 0.000 | 0.000-0.203 | 0.001-0.007 | 0.000 |
| *Notarius rugispinis* | 0.081-0.176 | 0.000 | 0.081 | 0.089-0.160 | 0.000 | 0.089 |
| *Notarius quadriscutis* | 0.000-0.161 | 0.000-0.004 | 0.000 | 0.000-0.201 | 0.003-0.010 | 0.000 |
| *Bagre bagre* | 0.104-0.182 | 0.000-0.002 | 0.102 | 0.156-0.203 | 0.001-0.012 | 0.144 |
| *Cathorops agassizii* | 0.010-0.187 | 0.000-0.006 | 0.004 | 0.016-0.183 | 0.000 | 0.016 |
| *Cathorops spixii* | 0.010-0.179 | 0.000-0.006 | 0.004 | 0.016-0.187 | 0.001-0.007 | 0.009 |
| *Notarius grandicassis* | 0.081-0.187 | 0.000-0.014 | 0.067 | 0.089-0.182 | 0.000-0.006 | 0.083 |
| *Sciades couma* | 0.039-0.158 | 0.000-0.006 | 0.033 | 0.032-0.174 | 0.000-0.007 | 0.025 |
| *Sciades herzbergii* | 0.041-0.156 | 0.000 | 0.041 | 0.047-0.174 | 0.000-0.004 | 0.043 |
| *Sciades parkeri* | 0.048-0.153 | 0.000 | 0.048 | 0.055-0.181 | 0.000 | 0.055 |
| *Sciades passany* | 0.037-0.156 | 0.000 | 0.037 | 0.032-0.169 | 0.000 | 0.032 |
| *Sciades proops* | 0.041-0.150 | 0.000-0.008 | 0.033 | 0.047-0.176 | 0.000 | 0.047 |
